# Purulent Vulvo-Vaginitis in Little Girls

**Published:** 1892-02

**Authors:** 


### PURULENT VULVO-VAGINITIS IN LITTLE GIRLS.

Chaumier (*Poitu Medical*, October, 1891) calls attention to the purulent vulvo-vaginitis of little girls, which according to certain authors, owes its origin to blennorrhagic infection. The writer holds that the disease may also be due to other causes, notwithstanding the presence in the discharge of the gonococcus of Neisser. The disorder is contagious, and is of more frequent occurrence in large cities than in the country. It always exhibits the same aspect, characterized by a thick purulent discharge, agglutination of the great lips and retraction of the vulva and vagina, but without complicating the urethral canal. Vulvo-vaginitis is often accompanied with a slight feverish reaction, loss of appetite, frequent and painful urination, the pain being due solely to the passage of the liquid over inflamed parts. In a case related by the author the disease was complicated with an arthritis of the knee-joint. If the malady is left to itself it may last for a long time, but under proper treatment it will generally yield in from two to two and a half months. The treatment consists in the external applications of lotions and in the vaginal injections of corrosive sublimate, followed by the introduction of salol pencils. Salol must also be given internally.—*Annals of Gynœcology and Pœdiatry.*

